# Organic Fluorescent Probes for Monitoring Micro-Environments in Living Cells and Tissues

**DOI:** 10.3390/molecules28083455

**Published:** 2023-04-14

**Authors:** Rui Yang, Tao Zhu, Jingyang Xu, Yuang Zhao, Yawei Kuang, Mengni Sun, Yuqi Chen, Wei He, Zixing Wang, Tingwang Jiang, Huiguo Zhang, Mengmeng Wei

**Affiliations:** 1 School of Electronics and Information Engineering, Changshu Institute of Technology, Changshu 215500, China; yangruithrive@163.com (R.Y.);; 2 Department of Key Laboratory, The Second People’s Hospital of Changshu, the Affiliated Changshu Hospital of Nantong University, Changshu 215500, China

**Keywords:** micro-environment, organelles, organic probes, fluorescence imaging, cells and tissues

## Abstract

As a vital parameter in living cells and tissues, the micro-environment is crucial for the living organisms. Significantly, organelles require proper micro-environment to achieve normal physiological processes, and the micro-environment in organelles can reflect the state of organelles in living cells. Moreover, some abnormal micro-environments in organelles are closely related to organelle dysfunction and disease development. So, visualizing and monitoring the variation of micro-environments in organelles is helpful for physiologists and pathologists to study the mechanisms of the relative diseases. Recently, a large variety of fluorescent probes was developed to study the micro-environments in living cells and tissues. However, the systematic and comprehensive reviews on the organelle micro-environment in living cells and tissues have rarely been published, which may hinder the research progress in the field of organic fluorescent probes. In this review, we will summarize the organic fluorescent probes for monitoring the microenvironment, such as viscosity, pH values, polarity, and temperature. Further, diverse organelles (mitochondria, lysosome, endoplasmic reticulum, cell membrane) about microenvironments will be displayed. In this process, the fluorescent probes about the “off-on” and ratiometric category (the diverse fluorescence emission) will be discussed. Moreover, the molecular designing, chemical synthesis, fluorescent mechanism, and the bio-applications of these organic fluorescent probes in cells and tissues will also be discussed. Significantly, the merits and defects of current microenvironment-sensitive probes are outlined and discussed, and the development tendency and challenges for this kind of probe are presented. In brief, this review mainly summarizes some typical examples and highlights the progress of organic fluorescent probes for monitoring micro-environments in living cells and tissues in recent research. We anticipate that this review will deepen the understanding of microenvironment in cells and tissues and facilitate the studies and development of physiology and pathology.

## 1. Introduction

Keeping the micro-environment healthy in cells and tissues is of vital importance to organisms. Multiple biological processes require a normal microenvironment, including viscosity, pH values, polarity, and temperature, to achieve basic life activity. As an important microenvironment, viscosity could control metabolic processes and diffusion-mediated processes at the cellular level, such as intra/intercellular signal transduction, the interactions of biomolecules, and proliferation of nitrogen species (RNS), etc. [1,2,3]. Moreover, pH is also a vital parameter in a number of bio-processes, including endocytosis, apoptosis, ion transport and proliferation [4,5]. Thirdly, as an indicator of hydrophilicity/ hydrophobicity, polarity is generally sensitive to solvents, so this kind of probe is named solvatochromic dyes [6,7]. The physiological process, such as enzyme-based catalysis and lipid composition, is closely associated with polarity [8,9]. Finally, various physiological and pathological states are in company with the variation of temperatures, so the temperature could reflect the state of cells and tissues [10,11]. Conspicuously, the microenvironment is of vital importance to biological organisms. The disorder would cause malfunctions in cells and tissues, and further promote the development of diseases. Thus, the exploitation of powerful tools for visualizing microenvironment in organisms is in urgent need.

As a vital constituent part in cells, organelles participate in various biological processes [12,13]. So, the research on visualizing the microenvironment in organelles is crucial for biologists and pathologists, though there are already some physical methods used for measuring environments, such as viscometer, thermometer, and pH meter [14,15,16]. However, they are used to measure the environments of macroscopic objects or aqueous solutions. Nowadays, various methods have been exploited to monitor microenvironment changes, including the electrochemical method, nuclear magnetic resonance (NMR), and spectral analysis and chromatography analysis [16,17,18]. However, many of these techniques required complicated sample preparation, and some techniques would destroy the biological samples [16,17]. So, these techniques could not be applied in the living biological systems. Moreover, the tools for measuring polarity are quite rare [7]. The fluorescent method assisted with proper fluorescence probes could in situ and real-time visualize the microenvironment changes, and it was widely applied due to the low cost, high sensitivity, nondestructive test, imperial spatiotemporal resolution, and simple operations [4,19,20,21]. Consequently, the fluorescent probes suitable for monitoring microenvironment in living cells and tissues have been widely explored.

**Figure 1:**
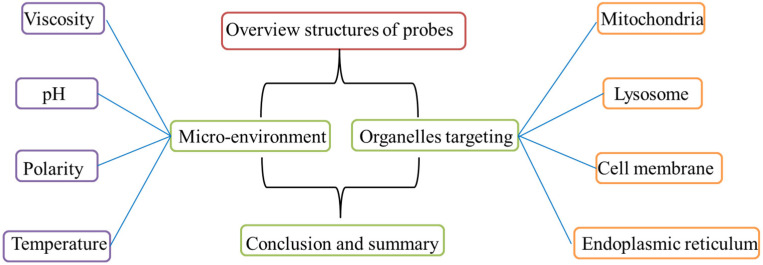
The description structures in this review.

Over recent years, the probes used for visualizing organelle environment has aroused researchers’ attention. Therefore, a large quantity of fluorescence probes was developed to monitor the microenvironment in organelles. However, the comprehensive and critical reviews about the visualizations of microenvironment in organelles were quite rare. So, in this review, we systematically summarize the microenvironment-sensitive probes for imaging organelles. To grasp the topic, the review was categorized into four common classes (viscosity, pH values, polarity, and temperature) according to the diverse physical properties, and each class was divided into four membrane organelles (mitochondria, lysosome, endoplasmic reticulum, cell membrane) in Figure 1. In each category, the design strategies, sensitive mechanisms, organelle targeting, and the biological applications were displayed and discussed. Moreover, the advantages and disadvantages between “off-on” probes and ratiometric probes were also elaborated. Finally, the brief summary and development tendency in this field were discussed. We anticipated that this review would be useful for further understanding this area, and it would provide useful methods for the construction of microenvironment probes, which would greatly promote the development in biology.

## 2. Organic Fluorescent Probes for Monitoring Viscosity

Viscosity is a paramount cell microenvironment, which influences various cellular processes and interactions of biological molecules, such as signal transduction, protein aggregation, membrane fusion and so on [22,23,24]. The abnormal changes of viscosity could disturb the normal physiological functions of biological systems, inducing various pathologies and diseases, such as hypertension, diabetes, and Alzheimer’s disease [25,26,27,28]. Thus, monitoring viscosity profile in cells and tissues is of vital importance in uncovering cellular function and biology roles in health and disease. Nowadays, a large variety of methods was developed to measure viscosity, such as rotary viscometer, capillary viscometer and magnetic resonance elastography (MRE), etc. However, their ranges of applications are limited, and the imaging resolution is low [29,30,31,32]. In view of this circumstance, organic fluorescent probes for monitoring viscosity occur due to their fast-response, high sensitivity, and non-invasive to living cells and tissues [33,34].

### 2.1. Organic Probes for Mitochondrial Viscosity

Mitochondrial viscosity plays an important role in various life activities, such as the reflection of tricarboxylic cycles and respiratory state [35]. Moreover, the increase of mitochondrial viscosity may induce dysfunctions, atherosclerosis, even malignancy in organisms [33,34]. So, monitoring mitochondrial viscosity is of vital importance to physiology and pathology.

**Figure 2:**
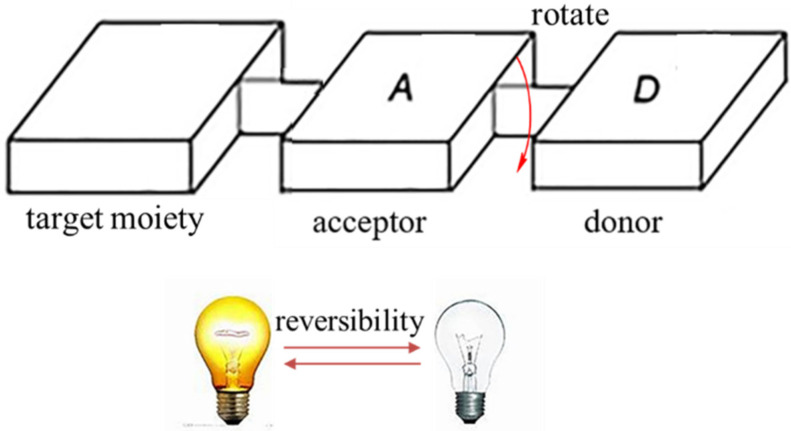
General composition and fluorescent mechanism of rotors.

As a typical fluorescence sensor of viscosity, molecular rotors could be manipulated by the molecular excited state planarization, and it was used to monitor mitochondria viscosity [36]. As shown in Figure 2, the high rotational freedom makes the rotors have “on-off” property. Generally, molecular rotors are composed of three parts: an electron-acceptor, an electron-donator, and a π-conjugated linking moiety [37]. In addition, the electron could transfer from the electron-donator to the electron-acceptor via π-conjugated structure when the planar conformation occurs. So, they may have strong fluorescence when the restraint of intramolecular rotational relaxation between donor−acceptor occurred. However, it will not fluoresce when intramolecular rotational relaxation occurs [38]. These properties made rotors more useful for visualizing viscosity changes.

**Figure 3:**
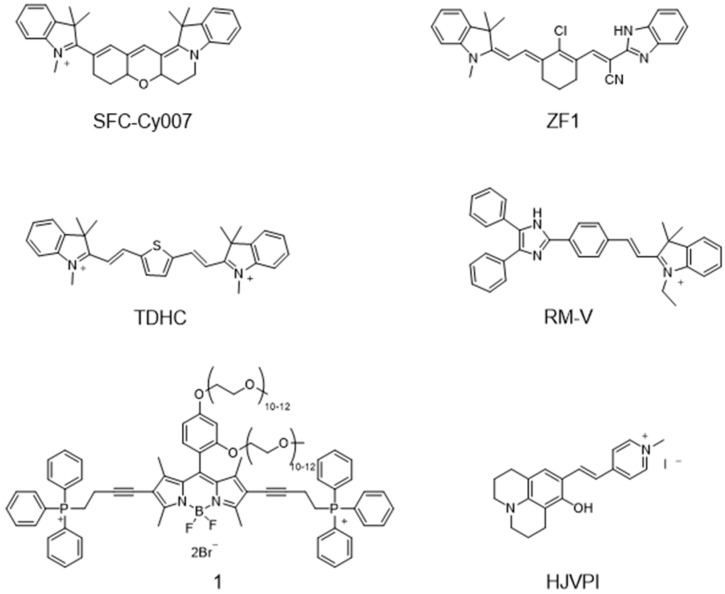
Typical fluorescent probes for visualizing mitochondrial viscosity.

In view of the excellent properties of the fluorescent rotor, a mass of fluorescent probes sensitive to viscosity was developed. Park et al. have exploited a viscosity-sensitive mitochondrial fluorescence probe (SFC-CY007, Figure 3), which is able to visualize viscosity changes in far-infrared channels [39]. Moreover, the high photostability and brightness enable SFC-CY007 to be used to visualize mitochondria-related diseases. Zhang et al. have developed a novel near-infrared fluorescent probe with a large Stokes shift, and the probe was used to observe mitochondrial viscosity because it is sensitive to viscosity (ZF1, Figure 3A) [40]. Baek et al. have synthesized a viscosity-sensitive fluorescent probe with good stability for high-fidelity imaging of mitochondrial transport, and it was used to real-time visualize the viscosity changes of the motor neuron mitochondria (TDHC, Figure 3) [34]. Interestingly, the rapid transport of tubular mitochondria was also clearly visualized. Lin group have developed a viscosity-sensitive probe (RM-V, Figure 3) for visualizing mitochondrial viscosity changes [41]. Sui et al. developed a fluorescent probe, which was consisted of BODIPY, TPP and PEG (probe 1, Figure 3) [42]. The probe has good water solubility and could target mitochondria excellently. Zhang et al. developed a red emissive mitochondrial probe (HJVPI, Figure 3) with viscosity-sensitive properties to achieve the visualization of mitochondria with high fidelity [38]. This kind of probe is typical for visualizing mitochondrial viscosity, but these probes are not able to quantify mitochondrial viscosity. Thus, these studies of quantifying mitochondrial viscosity are still in progress.

**Figure 4:**
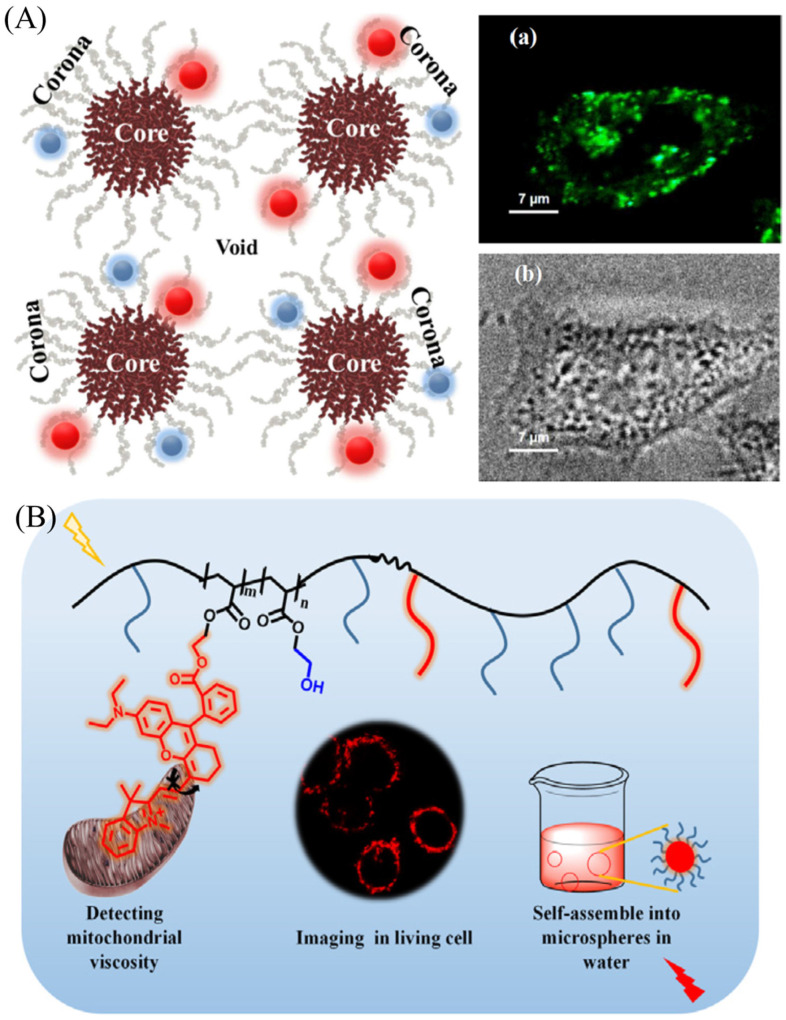
(**A**) Schematic figure of a triblock copolymer Pluronic F127 and the fluorescent images in Hela cells. (**a**) Confocal images of Hela cells stained with Pluronic F127. (**b**) DIC (Differential Interference Contrast) microscope pictures of HeLa cells. Reproduced from Ref. [43] with permission of the Wiley, copyright 2020. (**B**) The structure of amphiphilic copolymer fluorescent probe (PP) and their applications in detecting mitochondrial viscosity. Reproduced from Ref. [44] with permission of Elsevier, copyright 2021.

In addition, the organic nano-probes were also exploited for visualizing viscosity. Mukherjee et al. developed fluorescent Gold Nanoclusters for probing viscosity of HeLa Cells (Figure 4A) [43]. Yan et al. have developed an amphiphilic copolymer fluorescent probe (PP) consisting of the hydrophilic 2-hydroxyethyl acrylate and hydrophobic rhodamine fluorophore for detecting mitochondrial viscosity (Figure 4B) [44]. PP could self-assemble as nanospheres to monitor viscosity changes, and it exhibits excellent biocompatibility for visualizing viscosity changes in Hela cells. The polymers suitable for visualizing viscosity changes are still rare due to their bigger molecular structure, and the relative research is being conducted.

**Figure 5:**
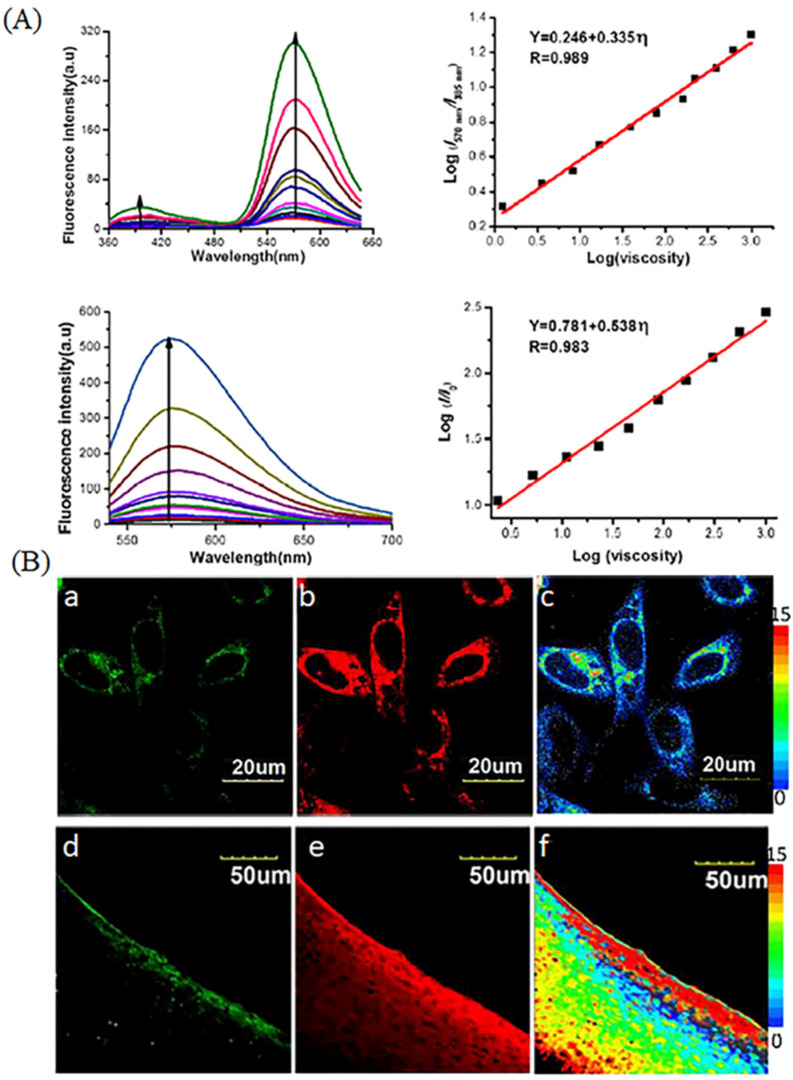
(**A**) Fluorescence changes and the linear response in the spectra of Qca-Cy2 in the solvent viscosity. (**B**) Fluorescence images of Qca-Cy2 in living HeLa cells and rat hepar slice in two channels. (**a**,**d**): green channel picture; (**b**,**e**): red channel pictures; (**c**,**f**): ratio pictures of (**a**) and (**b**); (**a**–**c**): confocal pictures of HeLa cells; (**d**–**f**): confocal pictures of rat hepar slice. Reproduced from Ref. [46] with permission of the Elsevier, copyright 2018.

**Figure 6:**
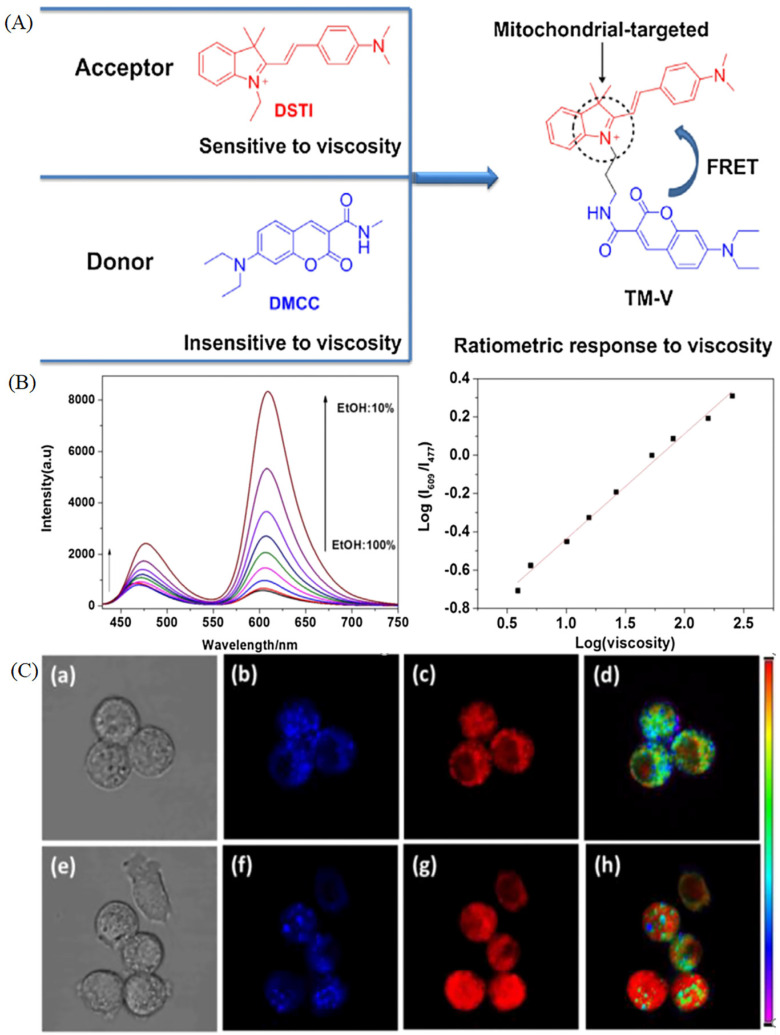
(**A**)The design principle of probe (TM-V) for ratiometric visualize mitochondrial viscosity in FRET mechanism. (**B**) The fluorescence intensity spectra of TM-V (10 μM) in the solvents of ethanol (**E**)–glycerol (**G**) mixtures under the exciting wavelength at 425 nm. (**C**) The bright field (**a**,**e**) and viscous fluorescence images (**f**–**h**) of TM-V (10 μM) in RAW 264.7 cells under normal stations (**b**–**d**) or treatment by PMA (**f**–**h**). Reproduced from Ref. [47] with permission of the Elsevier, copyright 2019.

In the study of viscosity, researchers found that the “off-on” probes have some drawbacks, such as the intracellular uptake, delivery, and retention of probes in diverse cell types, heterogeneities, sample thickness and light intensity and power dependency [45]. Compared with the “off-on” probes for visualizing mitochondria, the ratiomatric probe was developed due to the excellent properties, such as minimizing interference among analytes (heterogeneity, thickness, variations of dye loading and light intensity) [45]. In view of the excellent properties, ratiometric viscosity-sensitive probes were developed. Xu et al. have exploited a ratiometric two-photon fluorescence sensor (Qca-Cy2). The probe is sensitive to viscosity, and it could quantitatively detect the solution viscosity in spite of the complex environment, such as ions, common bio-macromolecules (Figure 5A). Moreover, the probes could ratiometric image HeLa cells and rat hepar slice using two photon microscopies (Figure 5B) [46]. Lin’s group has combined two fluorophores sensitive to viscosity to develop a ratiometric probe (TM-V, Figure 6A) for monitoring viscosity in dual color through FRET mechanism [47]. TM-V shows two emission peaks with the increase of solution viscosity, allowing for ratiometric visualizing localized viscosity in dual color (Figure 6B). Thus, the probe was used to visualize the increase of viscosity under the over-production of ROS (Figure 6C). This kind of probe is relatively rare due to the difficulty of dual-color fluorescence.

**Figure 7:**
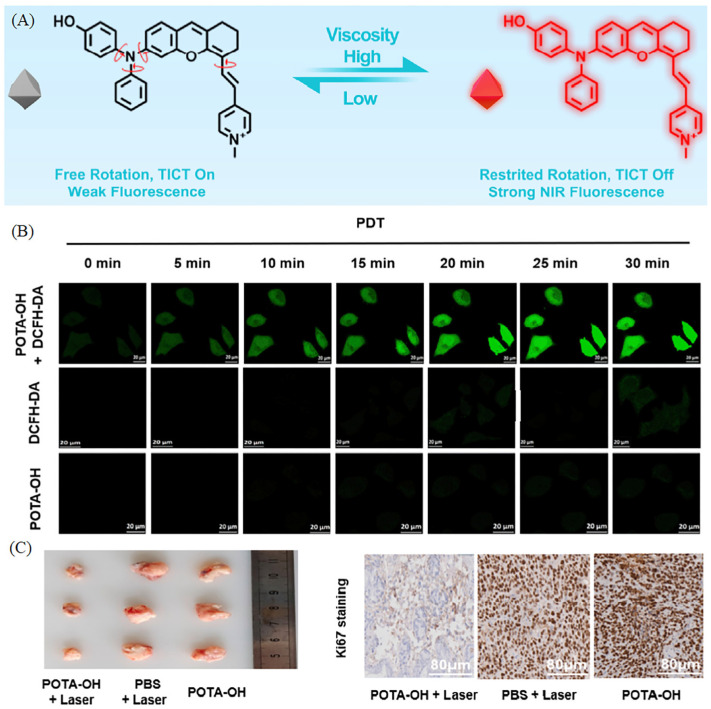
(**A**) The proposed mechanism of POTA-OH in response to viscosity. PDT experiment of probes in living cells (**B**) and tumor tissues (**C**). Reproduced from Ref. [48] with permission of the Elsevier, copyright 2022.

Recently, developing viscosity-sensitive mitochondrial probes suitable for visualizing the viscosity of cancer mice or diabetic mice has become a tendency. Dong et al. have reported a near-infrared mitochondrial probe sensitive to viscosity (POTA-OH) [48]. The viscosity of POTA-OH was measured in water/glycerol mixtures with the glycerol fraction (fG) from 0% (0.89 cP) to 99% (945 cP), and the fluorescent intensity was increased over 157-fold, indicting the high sensitivity to viscosity (Figure 7A). Moreover, the probe exhibited excellent ability of ROS generation upon laser irradiation (Figure 7B). So, the cancerous cells and tissues have been visualized by the high viscosity in mitochondria, and the high-efficient PDT treatment in tumor successfully proved that POTA-OH was potential for cancer diagnosis and treatment (Figure 7C). Zhou’s group has developed a viscosity-sensitive NIR mitochondrial probe for visualization of viscosity in living cells and diabetic mice model [49]. The fluorescent intensity was quite strong in the pancreas of diabetic mice, which indicates that the probe might be used for the visualizing the changes in pancreas. Nowadays, the viscosity-sensitive mitochondrial probes were further used for monitoring model mice, because the diseases of model mice are closely relative to viscosity. This kind of research has practical application values, and the research would prompt the development of medical science.

### 2.2. Organic Probes for Lysosomal Viscosity

As an important microenvironment parameter, lysosomal viscosity could reflect the status and function of lysosomes [50]. So, lysosomal viscosity is associated with the occurrence of various diseases, such as cancer [51]. Real-time visualizing dynamic alteration of lysosomal viscosity is meaningful for diagnosis of lysosome-related diseases and fundamental cell biology research [52]. Thus, visualizing lysosomal viscosity is of great significance.

**Figure 8:**
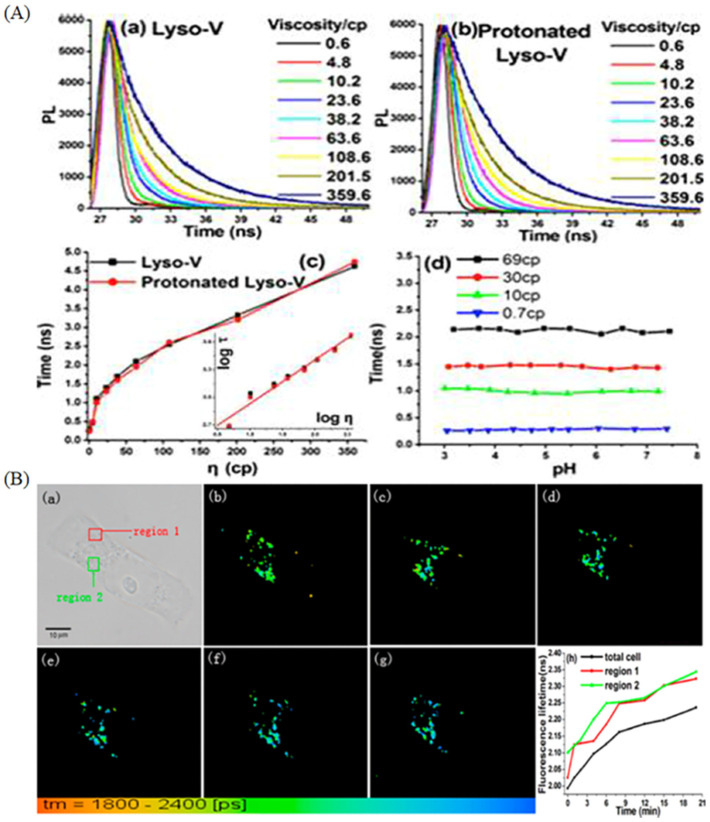
(**A**) Fluorescence lifetime spectra (**a**) of Lyso-V at the concentration of 2 μM and protonated Lyso-V (**b**) in at the concentration of 4 μM methanol/glycerol mixtures, and their Fluorescence lifetimes in diverse viscosity (**c**) and pH values (**d**). (**B**) Fluorescence lifetime imaging of Lyso-V in MCF-7 cells at diverse times after treatment with 5 μM dexamethasone and their corresponding fluorescence lifetimes. (**a**) DIC; (**b**–**g**). Fluorescence lifetime imaging at diverse time (0, 1, 2, 6, 8, 12 min). (**h**) the corrsponding fluorescence lifetime of different regins (1,2 and total cell) in diverse time. Reproduced from Ref. [52] with permission of the American Chemical Society, copyright 2013.

**Figure 9:**
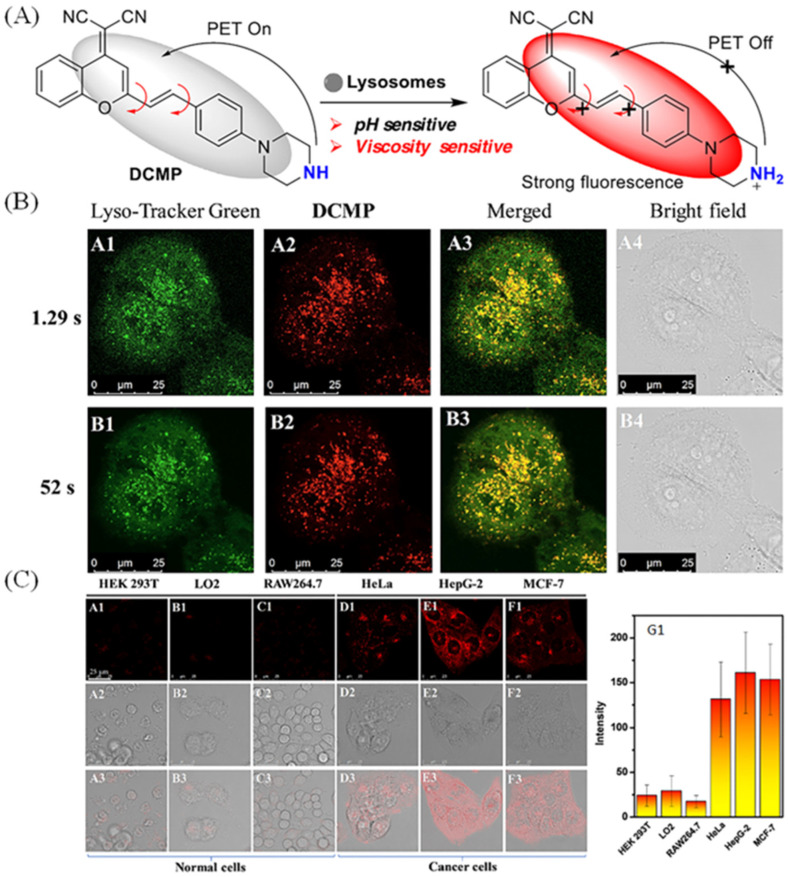
(**A**) The design mechanisms of DCMP sensitive to viscosity. (**B**) Co-staining fluorescence pictures of Lyso-Tracker Green (200 nM, **A1**, **B1**) and DCMP (100 nM, **A2**, **B2**) under different scanning speed (exposure time 1.29 s and 52 s). **A3**, **B3**: the merged pictures of **A1**, **A2** and **B1**, **B2**. (**C**) Fluorescent pictures of different types of cell line (cancer cells: HepG-2, HeLa, and MCF-7; normal cells: LO2, HEK 293 T, and RAW264.7) staining with DCMP (100 nM) and their relative intensity (**G1**) of different cells. **A1**–**F1**: confocal pictures of cells; **A2**–**F2**: DIC pictures; **A3**–**F3**: the corresponding pictures of **A1**–**F1** and **A2**–**F2**. Reproduced from Ref. [51] with permission of the Elsevier, copyright 2022.

In view of the advantages of fluorescence technology, a lot of fluorescent probes were exploited to monitor lysosomal viscosity. Deng et al. exploited a viscosity-sensitive fluorescent probe (Lyso-V) for visualizing lysosome through linking BODIPY rotor with a morpholine moiety [52]. The fluorescence lifetime of Lyso-V and protonated Lyso-V exhibited satisfactory viscosity-sensitive properties, and the lifetime increased from 0.30 to 4.62 ns with the increase of viscosity from 0.6 to 359.6 cP (Figure 8A). In addition, the lifetimes of neutral and protonated Lyso-V are identical with the increase of viscosity (Figure 8A). So, Lyso-V was applied to monitor the lysosomal viscosity changes through the FLIM technique after treatment with dexamethasone, and the average lifetime in Hela cells was enhanced from 1.99 to 2.24 ns, indicting the lysosomal viscosity changes from 67 to 85 cP (Figure 8B). To discriminate normal cells and tumor cells, Feng’s group developed a viscosity-sensitive probe (DCMP) due to the fact that tumor cells have larger viscous values [51]. The fluorescence intensity was connected with the PET mechanism and rotor properties, and it had weak fluorescence when the rotation and PET effect happened. When the reduced pH and the limited rotation occurred, the fluorescence intensity became strong (Figure 9A). In Figure 9B, in spite of the viscosity-sensitive property, the probe could visualize lysosomes clearer at different scanning speed (exposure time 1.29 s and 52 s) compared with Lyso-Tracker Green (a commercial lysosomal probe). Thus, the probe was used to discriminate between normal cells and tumor cells. In Figure 9C, the tumor cells have stronger fluorescence intensity compared with normal cells, further indicting the tumor cells are more viscous.

**Figure 10:**
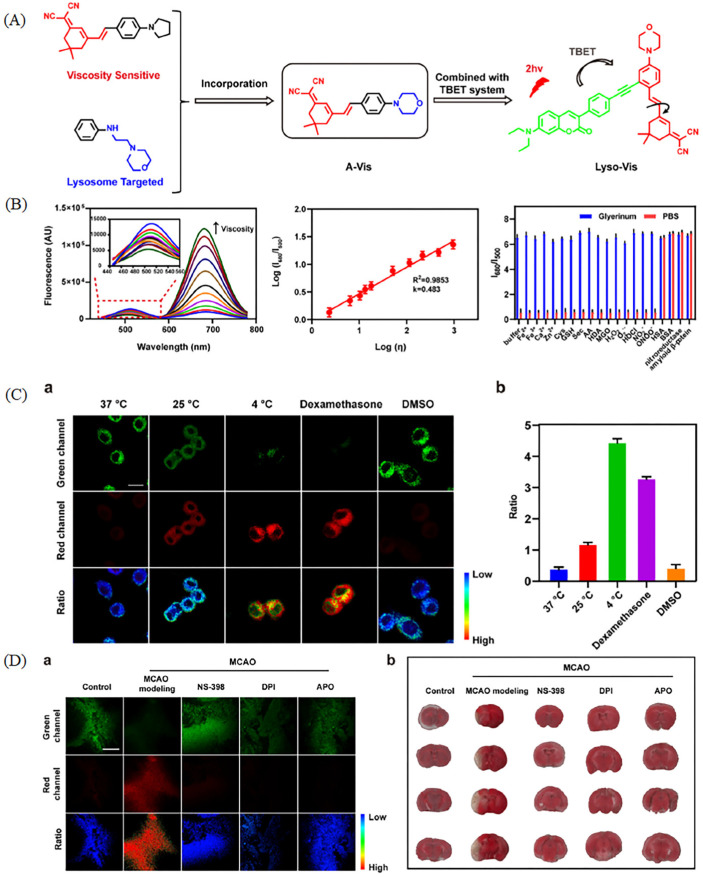
(**A**) Design strategy of Lyso–Vis with TBET mechanism. (**B**) Fluorescence spectra of Lyso–Vis in diverse viscosity and the corresponding linear relationship between log (I_672_) and log η; Fluorescence spectra of Lyso–Vis under diverse analyst solution. (**C**) Fluorescence images and the corresponding data of PC12 cells incubated with Lyso–Vis under different treatments (**a**) and the corresponding ratio (**b**), such as temperature, dexamethasone and DMSO. (**D**) Two-photon pictures (**a**) of mice brain slices stained with Lyso–Vis under diverse treatments: Control; MCAO modeling, NS-398; DPI; APO. (**b**) the corresponding mice brain slices pictures in (**a**). In addition, the corresponding TTC staining images of infarct regions reproduced from [53] with permission of Elsevier, copyright 2022.

In spite of the drawbacks of “off-on” probes stated above, the ratiometric fluorescent probes were exploited to visualize viscosity in dual colors. To achieve ratiometric visualization of lysosomal viscosity, Liu et al. have exploited a viscosity-responsive ratiometric two-photon probe (Lyso–Vis) with the through bond energy transfer (TBET) mechanism for tracking autophagy process [53]. The lysosome-targeted moiety and viscosity-sensitive moiety of Lyso–Vis were constructed with coumarin to achieve the TBET mechanism, which could improve the resolution of imaging (Figure 10A). Lyso–Vis could monitor viscosity in ratiometric manner, and it has excellent sensitivity and selectivity to viscosity (Figure 10B). Lyso–Vis was used to visualize viscosity changes in dual color in PC12 cells due to the excellent properties state above. In Figure 10C, the probe could monitor viscosity in ratiometric manner, so the probe was used to monitor autophagy in mice stroke models. The stroke model mice made by the middle cerebral artery occlusion (MCAO) method displayed higher viscosity, and the damage by stroke in cerebral tissue would be alleviated after treatment with inflammatory inhibitors (Figure 10D). This comprehensive research would prompt the development of medical science.

### 2.3. Organic Probes for Cell Membrane Viscosity

**Figure 11:**
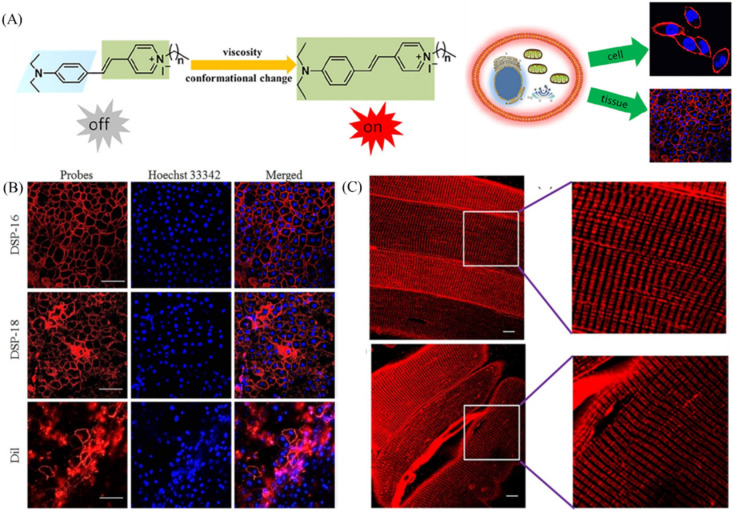
(**A**) Mechanisms of probes sensitive to plasma membrane viscosity and their applications. (**B**) Co-staining of viscosity-sensitive probes (DSP-16 and DSP-18), Dil (a commercial plasma membrane probe) with Hoechst 33342 (a commercial nuclei probe) respectively in hepatic tissues. (**C**) Confocal pictures of skeletal muscle tissues stained with DSP-16 and DSP-18. Reproduced from [59] with permission of the Elsevier, copyright 2021.

**Figure 12:**
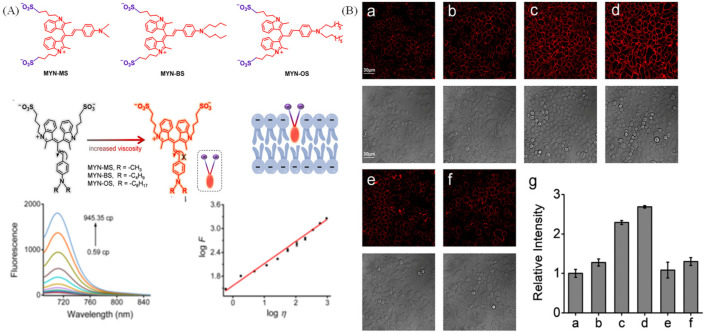
(**A**) Mechanisms of probes sensitive to plasma membrane viscosity and their fluorescence spectra in diverse viscous mixture. (**B**) Confocal images of macrophage-derived foam cells using various concentrations of ox-LDL (0 (**a**), 20 (**b**), 50 (**c**) or 100 (**d**) mg/mL), then stained with 10 mM MYN-BS. Images (**e**) and (**f**) are of cells pretreated with 100 mg/mL ox-LDL for 24 h in the presence of TMN355 (2 mM) and ezetimibe (10 mg/ mL). (**g**) the relative pixel intensities. Reproduced from [60] with permission of the Royal Society of Chemistry, copyright 2022.

As the front barrier of the cell, the cell membrane controls many bio-molecular interactions through selective transportation of substances into cells [54]. The cell membrane viscosity has many influences on drug delivery, drug diffusion and many physiological processes [55,56]. The abnormal alteration of cell membrane viscosity is associated with various diseases, such as diabetes, Alzheimer’s, atherosclerosis and cell malignancy [57,58]. Thus, the visualization of cell membrane viscosity is crucial for diagnose of relative diseases. Nowadays, cell membrane probes are always designed by linking long alkyl chain to rotors. Yu’s group has developed two cell membrane probes sensitive to viscosity for high-fidelity imaging cell membrane in living cells and tissues [59]. The two probes displayed typical rotor properties, showing “off-on” fluorescence to viscosity (Figure 11A). Compared with the commercial cell membrane probe (Dil) with larger molecular structure, the DSP-16 and DSP-18 could visualize cell membrane in hepatic tissues due to the excellent properties (Figure 11B). Interestingly, these two probes could clearly image transverse tubule (T-tubule), which exists in skeletal muscle tissues (Figure 11C). Ma’s group has developed NIR fluorescent probes (MYN-MS, MYN-BS and MYN-OS) for assessing cell membranes viscosity levels [60]. These probes have general plasma membrane structures, and they show viscosity-sensitive fluorescent properties (Figure 12A). In line with the good properties, the MYN-BS was introduced to visualize macrophage-derived foam cells induced by low-density lipoprotein (ox-LDL) with strong fluorescence intensity, and the fluorescence intensity of cells treated with TMN355 and ezetimibe (two inhibitors) are quite weak, indicting the increase of viscosity during cell foaming.

Although the probes stated above could visualize cell membrane viscosity, they could not quantify membrane viscosity. Thus, in the future, more probes could be developed to achieve the quantification of cell membrane viscosity, so as to provide effective guidance for viscosity-related diseases.

### 2.4. Organic Probes for Endoplasmic Reticulum Viscosity

Endoplasmic reticulum (ER) viscosity is one of vial parameters in cells and tissues, and the changes viscosity is caused by the accumulation of unfolded or misfolded proteins [61]. Thus, it is valuable to develop ER probes sensitive to viscosity to monitor viscosity changes.

**Figure 13:**
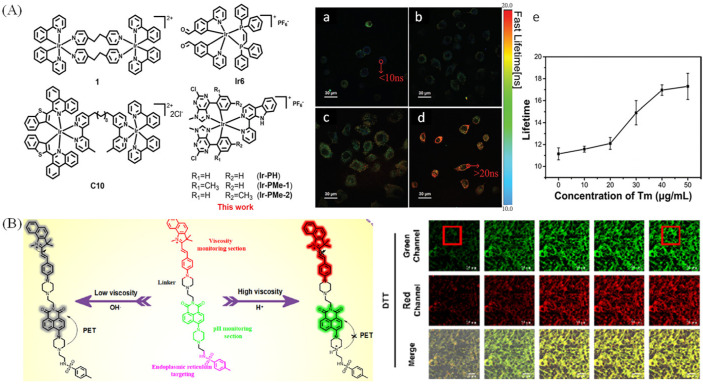
(**A**) Chemical structures of viscosity-sensitive iridium (III) complexes and the FLIM pictures of A549 cells stained with Ir−PH treated with Tm (0, **a**; 20, **b**; 30, **c**; 50, **d**; mg/mL); (**e**) the lifetime of cells under diverse concentration of Tm. Reproduced from 66 with permission of the Royal Society of Chemistry, copyright 2021. (**B**) The mechanisms of DSPI-3 sensitive to viscosity and pH, and dual-color visualize cells by dithiothreitol (DTT). Reproduced from [67] with permission of the American Chemical Society, copyright 2022.

Nowadays, the probes used for visualizing ER viscosity are usually designed by connecting rotor to ER targeting moiety. Kim et al. have exploited an ER membrane targeting probe (V-1) by linking BODIPY to coumarin with a long alkyl chain (n-C18) for monitoring ER viscosity [62]. With the aid of β-CD, V-1 could target ER in HeLa cells with dual color due to the two comprising fluorophores (BODIPY/coumarin), and they used the probe to study ER membrane fluidity induced by various ER stress. To solve the permeability problems of ER probes with long alkyl chain, Yu’s group developed a viscosity-sensitive probe with rotor properties for visualizing ER with high-fidelity [63]. The probe has excellent permeability, and it was used to real-time monitor ER-autophagy. Then, the fluorescence lifetime has aroused researchers’ attention to monitor of ER viscosity to improve the drawbacks based on intensity. Yu et.al have developed an iridium (III) complex sensitive to viscosity to visualize viscous changes with fluorescence lifetime imaging microscope (Figure 13A) [64]. Ir-PH enables to visualize ER viscosity due to the correction between lifetime and viscosity, and the slightly longer lifetimes indicated that increase of viscosity treated with Tunicamycin ™.

Recently, the viscosity changes of ER induced by ferroptosis has aroused researcher’s interest. Xiao et.al developed a new type of probe (L-Vis-1) by linking ER targetable group to a BODIPY rotor to sense viscosity with its fluorescence lifetime, and the real-time analysis of ER viscosity changes during different ferroptosis processes were achieved by L-Vis-1 and fluorescence lifetime imaging (FLIM) [65]. Kim group have developed a bimodal probe through combining Nile Red with BODIPY to monitor micropolarity and microviscosity of the endoplasmic reticulum [66]. Lin’s group exploited a probe sensitive to pH and viscosity, and it used the probe DSPI-3 to real-time reveal the changes of pH and viscosity of ER under the treatment of ferroptosis (Figure 13B) [67]. Due to dithiothreitol (DTT) could result in ER acidification and viscosity enhancement, they used DTT to visualize pH and viscosity changes. In Figure 13B, green and red fluorescence were significantly enhanced, indicating ER acidification and the increase of viscosity.

In addition, there are also some viscosity-sensitive probes for visualization of other organelles. Tian et al. have exploited two small organic molecules with optimized two-photon action cross-section. These probes are water-soluble, and they could ratiometric light up nucleic acid with two unique fluorescence [68]. Moreover, the two probes were utilized for two-photon visualizing nucleic acid in rat live tissue, and they displayed high signal ratio, good photostability, as well as excitation with NIR light, which made them have potential applications in biological and biomedical research. The multifunctional probes for visualization of viscosity and analytes in dual color are also a tendency in the environment-sensitive areas [69]. Above all, multiduty probes for monitoring viscosity in cells would be the tendency in the future.

## 3. Organic Fluorescent Probes for Monitoring pH

As a vital parameter in cells, the pH values are of vital importance to various life activities. Different organelles have discrepant pH values, such as mitochondria (pH~8), lysosome (pH:4.5–5.5), cytosol (pH:6.8–7.4) [15,70]. The abnormal pH values would lead to various diseases, such as cancer, Parkinson’s diseases and Alzheimer’s diseases, [71,72,73]. So it is crucial for visualizing pH values in organelles.

### 3.1. Organic Probes for Mitochondrial pH

**Figure 14:**
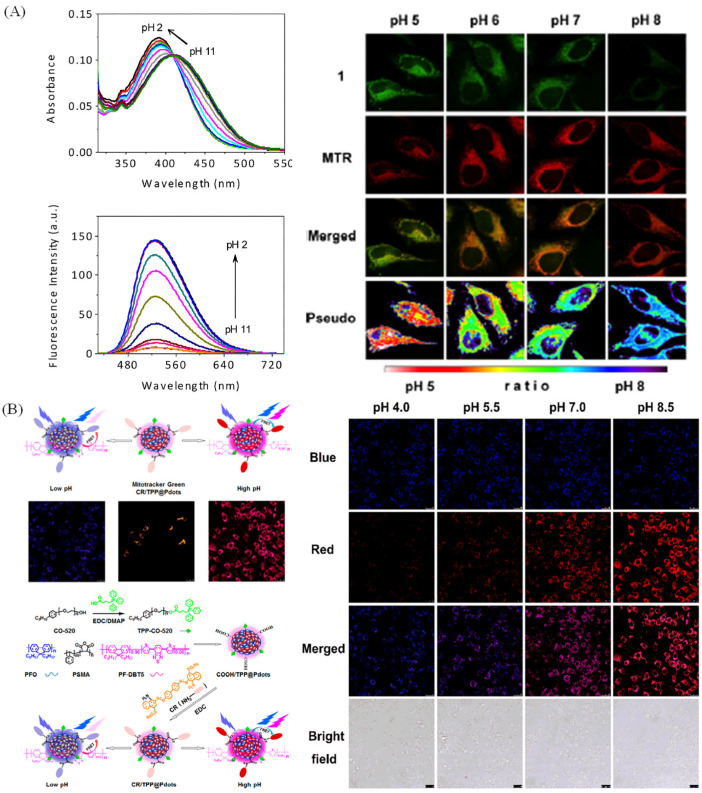
(**A**) Fluorescence spectra of in diverse pH solutions, and its fluorescence pictures in fixed cells. Reproduced from 80 with the American Chemical Society, copyright 2014. (**B**) Synthesis of CR/TPP@Pdots and its applications in HeLa cells with diverse pH values. Reproduced from [79] with permission of the American Chemical Society, copyright 2017.

The pH can reflect the states of various biological processes, including signaling, adenosine triphosphate production, cell cycle and death [74]. Thus, visualizing dynamic mitochondrial pH could understand the relative biological processes, and the measurement of pH values with optical method was exploited due to the excellent properties of optical method [75,76,77]. To visualize mitochondrial pH, Kim group have developed a mitochondrial probe (probe 1, Figure 14A) with “off-on” properties to pH, and it was applied to research pH in mitochondria. To subtly calibrate intracellular pH, HeLa cells were co-stained with probe 1 and MTR, and then the cells were fixed in buffer solutions with diverse pH. The fluorescence intensity of the probe gradually decreases with the increase of pH (Figure 14A), whereas the fluorescence intensity of MTR remained essentially unchanged. Moreover, the ratio of the green and red emission intensities in pseudocolored images demonstrated that the probe could visualize pH changes in mitochondria [78]. Gao et al. have provided a pH- sensitive functionalized Pdots (CR/TPP@Pdots) for monitoring mitochondrial pH values [79]. The Pdots could emit dual colors with a high selectivity, wide pH detection rang, fast response and good reversibility, and it was successfully used to dual-color image pH in the Raw 264.7 cells (Figure 14B). Nowadays, pH–sensitive probes for ratiometric visualizing mitochondrial pH have been developed, [80,81,82]. However, the nao-probes for ratiometric visualizing mitochondrial pH are relatively rare.

### 3.2. Organic Probes for Lysosomal pH

As an acid organelle (acidic environment of 4.5–6.5) in living cells, the lysosome could accelerate the metabolism of macromolecular, and it is associated with human diseases and cell ageing [20]. Thus, the anomalous fluctuations of lysosomal pH will lead to the disorder of normal cell activities, and cause diseases, including cancer [20,83].

**Figure 15:**
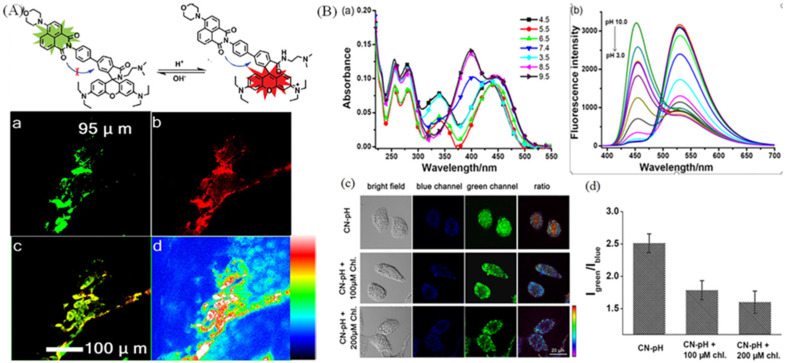
(**A**) Ratiometric response mechanism of lysosomal fluorescent probe NRLys to pH and the ratiometric imaging of mice muscle tissue slice. (**a**) Confocal pictures in green channel; (**b**) Confocal pictures in red channel. (**c**) Merged pictures of (**a**) and (**b**); (**d**) Ratiometric pictures of (**a**) and (**b**). Reproduced from [85] with permission of the American Chemical Society, copyright 2017. (**B**) Absorbance (**a**) and fluorescence (**b**) spectra of CN-pH in diverse pH solution, and the dual dolor images (**c**) of lysosomal pH changes in HeLa cells treated with 100 and 200 μM chloroquine. (**d**) The relevant ratios in (**c**).

Nowadays, the construction of probes for visualizing lysosomal pH is based on amine functional group with the protonation of the N atom or rhodamine group with ideal ‘‘off–on’’-type spirocyclic structure. Wang et al. have exploited a near-infrared probe (Lyso-hNR) for selectively tracking lysosomal pH values with rhodamine and morpholine structure [84]. Lyso-hNR is sensitive to pH due to the H^+^-induced ring opening, and it was used to track and detect lysosomal pH changes because of the rapid response, good reversibility, and high selectivity properties. Compared with the “off–on” type, the ratiometric probes were also exploited. Zhang group have developed a ratiometric fluorescent probe sensitive to pH for dual-channel imaging of lysosome by combining xanthane derivative and naphthalimide group [85]. The probe could emit two colors through FRET mechanism between xanthane and naphthalimide group in diverse pH, and it could ratiometric visualize mice muscle tissue slice in dual color due to the good permeability to tissue (Figure 15A). Lin’s group has developed a ratiometric lysosome-targeted fluorescent probe (CN-pH) based on ICT−PET−FRET mechanism for dual color visualizing lysosome (Figure 15B) [19]. CN-pH displayed dual-color emission in diverse solutions with different pH value, and it could visualize pH changes in Hela cells stimulated with chloroquine.

### 3.3. Organic Probes for Monitoring pH around Cell Membrane Surface

The cell surface is not only the boundary of a cell but also participates in a wide range of cellular activities, including the recognition of pathogens, extracellular matrix modeling, and so on [86,87]. Thus, visualizing the micro-environment around cell membrane surface is crucial for medical and biological research.

**Figure 16:**
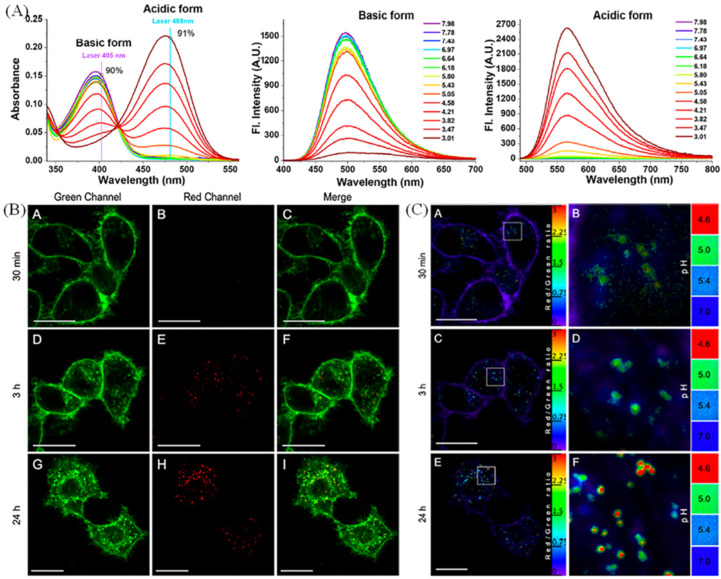
(**A**) Absorbance and fluorescence spectra of probe in solutions with diverse pH values. (**B**) Confocal images of KB cells incubated with Mem-pH (1μM, 1 h), and imaged after diverse time (30 min, **A**, **B**, **C**; 3 h, **D**, **E**, **F**; and 24 h, **G**, **H**, **I**;) in two channels and the ratiometric images (**C**) of KB cells after the same treatments (**A** 30 min; **C** 3 h; **E** 24 h; **B**, **D**, **F** are the enlarged picture of **A**, **C**, **E**). Reproduced from [89] with permission of the American Chemical Society, copyright 2022.

Nowadays, cell surface-related pH studies have achieved a certain development. Wang et al. have developed a cell-surface-anchored sensor based on FRET mechanism, and the sensor could detect extracellular pH due to the pH-sensitive i-motif structure [88]. The sensors could anchor on the cell surface by streptavidinbiotin interactions and achieved ratiometric readout extracellular pH. Collot group have develop a plasma membrane-targeted probe (Mem-pH) based on chromenoquinoline for measuring vesicular acidification [89]. Mem-pH has two absorbance and fluorescence peaks in diverse pH solutions, which could be used for dual-color imaging plasma membrane (Figure 16A). In Figure 16B, Mem-pH displayed two different targeting in two channels upon the formation of vesicles during endocytosis, and the probe could report the acidification of vesicles in ratiometric response to evaluate the vesicles’ pH.

### 3.4. Organic Probes for Endoplasmic Reticulum pH

As a vital parameter, the ER pH (pH = 7.2 ± 0.2) is critical for the regulation of physiological functions, such as protein sorting and targeting during secretion of resident chaperones, and the acidification of ER occurred as biosynthetic products approached their destination, which is closely related to diseases induced by ER stress [61,90,91,92].

**Figure 17:**
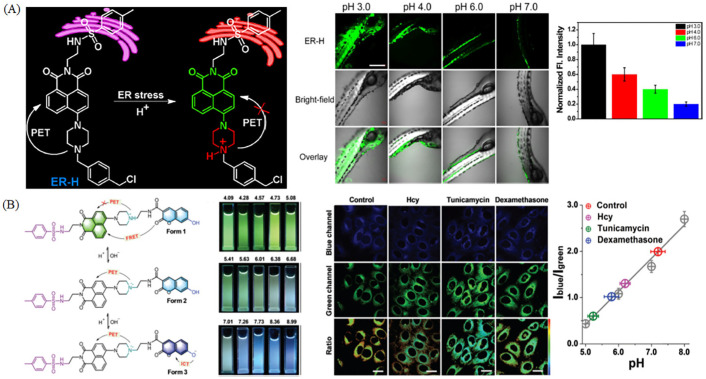
(**A**) Response mechanism of ER-H to pH, and the relative two-photon fluorescence imaging of zebrafish in diverse pH. The column diagram is the relative intensity changes. Reproduced from 95 with permission of the Elsevier, copyright 2018. (**B**) The pH-response mechanism and photographs of CNER-pH under irradiation at 365 nm; fluorescence images of HeLa cells stained with CNER-pH stimulated with Hcy, tunicamycin or dexamethanose, and the corresponding quantitative data. Reproduced from [94] with permission of the Royal Society of Chemistry, copyright 2019.

Nowadays, researchers exploit fluorescence probes for monitoring ER pH. Tang et al. have developed a pH-sensitive probe consisting of 4-methyl benzenesulphonamide moiety, thiol reactive benzyl chloride and 1,8-naphthalimide for visualization of ER acidification (Figure 17A) [93]. The probe ER-H has “off-on” property for pH, and it could visualize pH changes in Hela cells and zebrafish with diverse pH (Figure 17A). Lin group has developed a ratiometric fluorescent probe for the measurement of ER pH values based on ICT-PET-FRET mechanism (Figure 17B) [94]. The probe CNER-pH has dual-site for pH, and it could achieve dual-color emission based on ICT-PET-FRET mechanism. So CNER-pH was used to visualize HeLa cells stimulated with Hcy, tunicamycin or dexamethanose.

**Figure 18:**
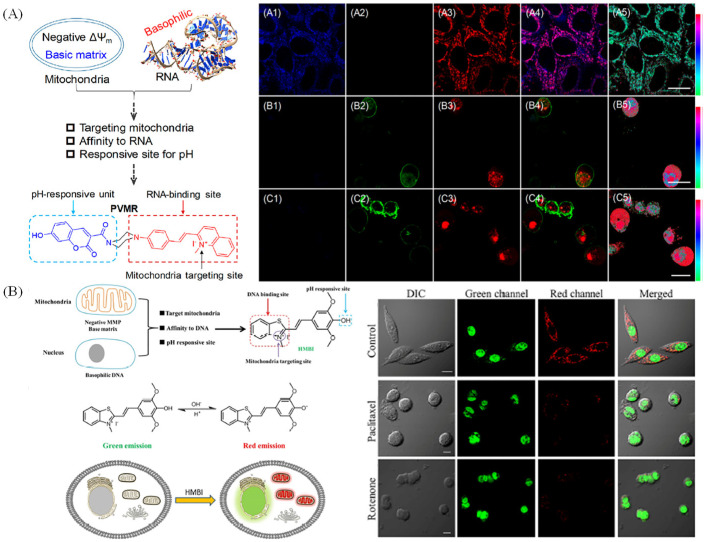
(**A**) Design routes of probe PVMR and the fluorescence and ratiometric images of HepG2 cells co-stained with PVMR and FITV−Annexin V after treatment with diverse culture medium (**A1−A5**), paclitaxel (**B1−B5**), or rotenone (**C1−C5**). **A1**–**C1**: blue channel pictures; **A2**–**C2**: green channel pictures; **A3**–**C3**: red channel pictures; **A4**–**C4**: Merged pictures of blue, green and red channels pictures. **A5**–**C5**: ratio pictures of blue and red channels. Reproduced from [95] with permission of the American Chemical Society, copyright 2019. (**B**) Design strategy of probe HMBI and their applications in apoptosis. Reproduced from [96] with permission of the American Chemical Society, copyright 2021.

Recently, single probes for visualizing two organelles in dual color were developed utilizing the discrimination of diverse organelles. Lin group have developed a pH-sensitive probe with dual color visualization of mitochondria and RNA, and the probe PVMR could target to mitochondria and release to apoptosis, due to the relationship properties between mitochondria and RNA [95]. PVMR was used to ratiometric monitor the cell apoptosis induced by drug treatment (Figure 18A). Yu group exploited a pH-sensitive probe for dual-color visualizing nuclei and mitochondria simultaneously (Figure 18B) [96]. The probes which could dual color visualize two organelles with diverse mico-environments is the tendency in this area because a lot of life activities are achieved by the cooperation of organelles.

## 4. Organic Fluorescent Probes for Monitoring Polarity

Among various microenvironments, cell polarity is a significant factor to reflect the state of cells, and cells also need to keep normal polarity to maintain specific functions [7]. Most cellular activities, such as local membrane growth and differentiation, may lead to the dynamic changes of polarity [97,98]. Therefore, the abnormal changes of polarity could be related to abnormal cellular activities.

### 4.1. Organic Probes for Mitochondrial Polarity

**Figure 19:**
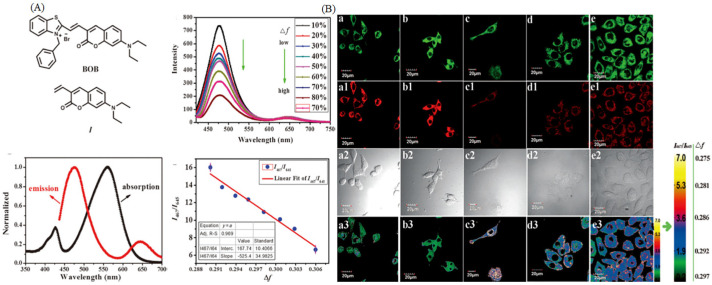
(**A**) Molecular structures, normalized absorption and emission of BOB. (**B**) Confocal of difference type of cells (cos-7 cells **a1**–**a3**, RAW 264.7 cells **b1**–**b3**, HeLa cells **c1**–**c3**, HepG2 cells **d1**–**d3**, and MCF-7 cells **e1**–**e3**) incubated with BOB and the ratio images. Green channel pictures: **a**–**e**; Red channel pictures: **a1**–**e1**; DIC pictures: **a2**–**e2**; Ratio pictures: **a3**–**e3**. Reproduced from [100] with permission of the Wiley, copyright 2015.

Mitochondria are a kind of dynamic organelle for providing energy for cellular activities, and the polarity of mitochondria is a vital indicator for abnormal mitochondrial functions. Yin et al. have developed a probe sensitive to polarity with “off-on” properties, and they used it to study the polarity changes in mitophagy with a commercial autophagy tracker, MDC (dansylcadaverine) [99]. Moreover, NIR-BT-P could distinguish normal cells from cancer cells, and it could observe the occurrence of mitophagy during heart starvation. Fan et al. have developed a ratiometric polarity-sensitive probe (BOB, Figure 19A), the probe has two absorption maxima (426 nm, 561 nm) and two emission maxima (467 and 642 nm) [100]. BOB could target mitochondria regardless of mitochondrial membrane potential (MMP). Thus, it was used to dual color visualize diverse types of cells. So, it was used to dual color visualize diverse types of cells due to the different polarity between normal and cancer cells (Figure 19B). Notably, dual-color polarity-sensitive probes are still rare, and further research is conducting around this area.

### 4.2. Organic Probes for Lysosomal Polarity

Lysosome is a vital organelle with digestion function, and the polarity of lysosome is always changing [101]. In many cellular activities involving lysosome membrane permeabilization and lysosome storage disorder, the various enzymatic reactions would be affected, leading to the alteration of lysosomal polarity. So, the visualization of polarity inside lysosome is crucial [101].

**Figure 20:**
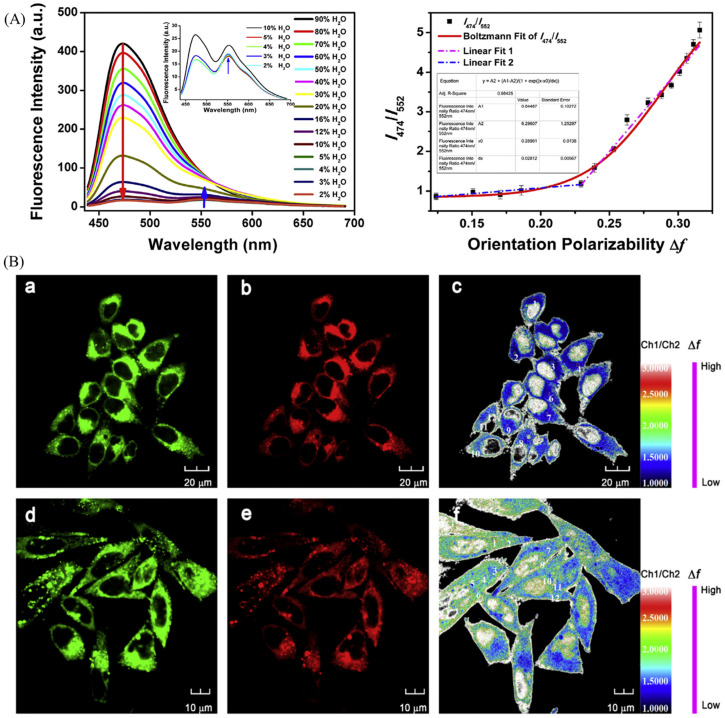
(**A**) Fluorescence spectra of NOH in 1,4-dioxane with diverse ratio of H_2_O and their fit curve of I_474_/I_552_ (**B**) Confocal fluorescence and ratiometric fluorescence images of MCF-7 cells stained with NOH, the experimental group are incubated with sucrose (80 mM, 10 min) to induce lysosomal storage disorders. Control groups: **a**–**c**; Experimental group: **d**,**e**; Green channels: **a** and **d**; Red channels: **b** and **e**; Ratio picutres: **c** and **f**. Reproduced from [103] with permission of the Elsevier, copyright 2019.

Dong et al. have developed a polarity-specific fluorescent probe (CMP) with lysosomal targeting. The probe is functional with coumarin (polarity sensitive motif) and morpholine (lysosomal targeting motif), and CMP was used to real-time monitoring the polarity changes of lysosome [102]. As for the ratiometric lysosomal probe, Peng group developed a lysosomal polarity probe, NOH, with dual emission colors for visualization of lysosome [103]. NOH displayed ratiometric properties in the mixture of 1,4-dioxane and H_2_O with diverse polarity (Figure 20A), and then it was used to lysosomal storage disorders. In Figure 20B, the pictures of MCF-7 cells with lysosomal storage disorders showed the increase of the polarity with the colors from glaucous from mazarine.

### 4.3. Organic Probes for Cell Membrane Polarity

Bio-membranes are the basis for various compartments in live cells, so visualizing their lipid organization is helpful for studying cell status and activity [6]. Polarity is a vital parameter in cell membrane, and it is the index of cell membrane status. Therefore, it is of vital importance for researchers to visualize cell membrane polarity.

**Figure 21:**
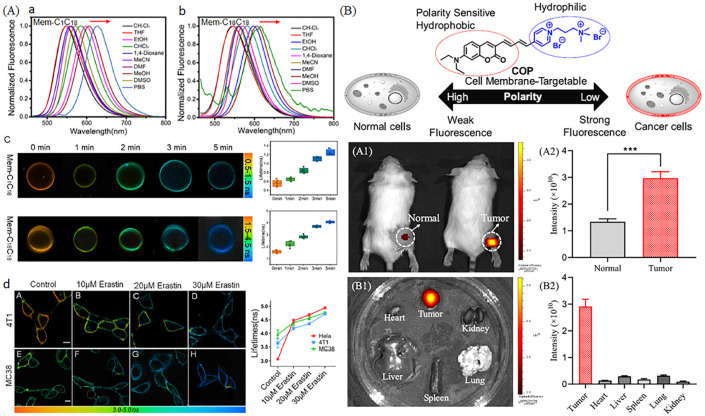
(**A**) Fluorescence spectra of Mem-C1C18 (**a**) and Mem-C18C18 (**b**) in several different polarity solvents, and the GUVs pictures (**c**) with a certain period of lipid peroxidation. (**d**) Pictures of 4T1 and MC38 cells incubated with Erastin. Reproduced from 106 with permission of the American Chemical Society, copyright 2022. (**B**) Mechanisms of probe COP and their applications. (**A1**) Fluorescence pictures of normal and tumor rats stained with COP and the corresponding fluorescence intensity (**A2**). (**B1**) Fluorescence organ pictures of tumor rats stained with COP and the corresponding fluores-cence intensity (**B2**). *** *p* (statistical significance) = 0.0004. Reproduced from [105] with permission of the American Chemical Society, copyright 2022.

Recently, various polarity-sensitive probes for visualizing cell membrane have been exploited. Xiao et al. have developed polarity-sensitive probes (Mem-C1C18 and Mem-C18C18) with membrane-specific properties for visualizing ferroptosis [104]. Mem-C1C18 and Mem-C18C18 displayed the environmental polarity in diverse solves, and the average fluorescence lifetime of GUVs (Lo and Ld) displayed an upward using FLIM in spite of the difference of lifetime response (Figure 21A). Then, the probes were used to study ferroptosis, and the fluorescence lifetime was increased from 3.00 to 4.93 ns in HeLa cells after treating with Erastin, a typical ferroptosis inducer, indicting probe could visualize polarity of cell membrane in ferroptosis process (Figure 21A). Feng et al. have developed a cell membrane with polarity-sensitive properties for visualizing polarity of tumor cell membrane [105]. The probe consisted of polarity sensitive coumarin and the hydrophilic cation salt, and it was used to discriminate the polarity of normal cells and cancer cells (Figure 21B). Moreover, the probe could emit strong red fluorescence in tumor tissues compared with normal tissues, indicting the lower polarity of cancer cell membranes.

### 4.4. Organic Probes for Endoplasmic Reticulum Polarity

**Figure 22:**
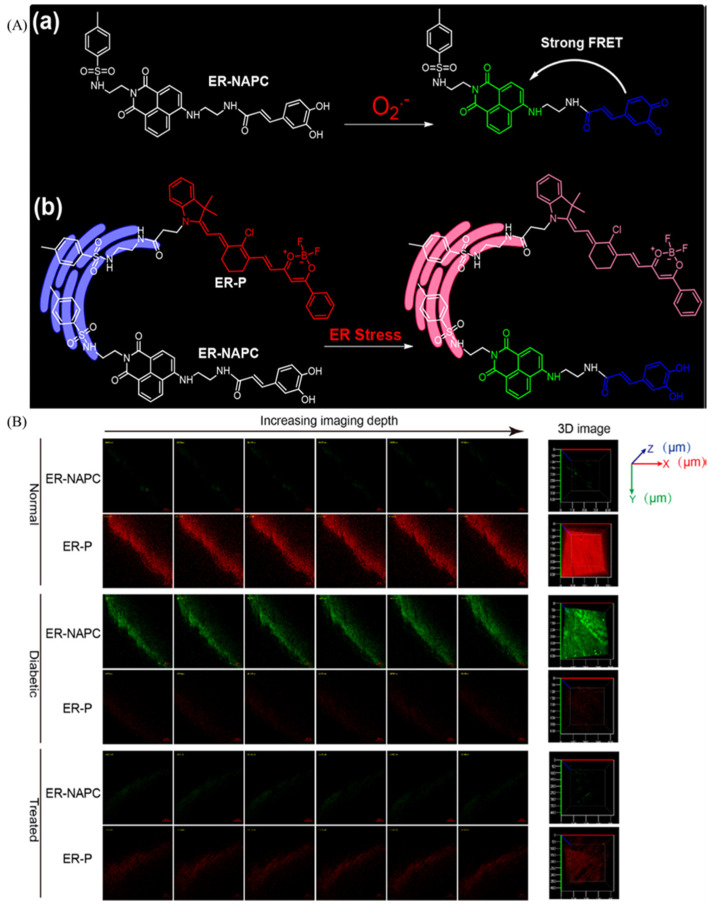
(**A**) Structure of ER-NAPC and O_2_^•−^ (**a**) and polarity (**b**) sensing mechanism. (**B**) Confocal pictures of endogenous polarity and O_2_^•−^ in myocardial tissues stained with ER-NAPC and ER-P. Reproduced from [107] with permission of the American Chemical Society, copyright 2018.

As an organelle with a large membrane structure, the endoplasmic reticulum is of vital importance to cells, and the polarity of endoplasmic reticulum needs to be visualized. Due to the urgent need for visualizing the changes of endoplasmic reticulum polarity, various probes were exploited. Smith et al. have developed an endoplasmic reticulum (ER) targeting probe through two coumarin moiety [106]. Tang et al. reported a polarity-sensitive probe for simultaneous visualization of superoxide anion and polarity [107]. ER-P was sensitive to polarity, and ER-NAPC could recognize superoxide anions (Figure 22A). Thus, the difference of concentration and polarity in normal, diabetic or treated diabetic mice were disclosed by the two probes. In Figure 22B, the normal tissues show red emitting colors, and the green fluorescence color was visualized in diabetic mice, which indicates the elevated polarity and superoxide anion of myocardial tissue in diabetic mice.

## 5. Organic Fluorescent Probes for Monitoring Temperature

Temperature is a crucial index in many pathological states and physiological activities [108], and the warm-blooded organisms should maintain nearly constant internal temperatures to live in a wide range of environments [109]. Moreover, the measurement of temperature is also of vital importance in engineering science [110,111,112]. Thus, visualizing temperature in living cells and tissues is necessary for diagnose of diseases and the applications of engineering science.

### 5.1. Organic Probes for Mitochondrial Temperature

**Figure 23:**
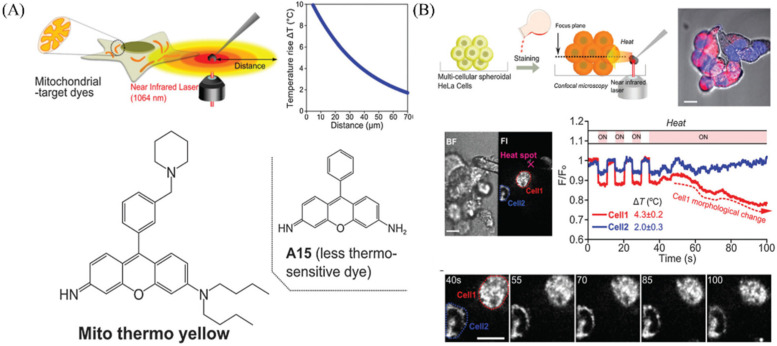
(**A**) The imaging systems to visualize temperature sensitivity and structure of probe Mito thermos yellow. (**B**) Monitoring the temperature with probe Mito hermos yellow, and the images and time course of fluorescence intensity in multi-cellular spheroidal HeLa cells. Reproduced from [114] with permission of the Royal Society of Chemistry, copyright 2015.

Mitochondrial temperature plays a vital role in cells and tissues, and recent studies suggest that cancer cells have relatively hot mitochondria, which indicates that the active respire of mitochondria in cancer cells [10,113]. Chang et al. have reported a small molecule mitochondria-targeted fluorescent probe (Mito hermos yellow) with rosamine structure for monitoring temperature changes in mitochondria [114]. Mito hermos yellow had rosamine structure with good brightness and could be sensitive to temperature (Figure 23A). So, it was used to monitor temperature changes in mitochondria, and it could visualize the morphological changes during continuous heating (Figure 23B). The probes sensitive to mitochondrial temperature are still rare, and the study is continuing.

### 5.2. Organic Probes for Lysosomal Temperature

**Figure 24:**
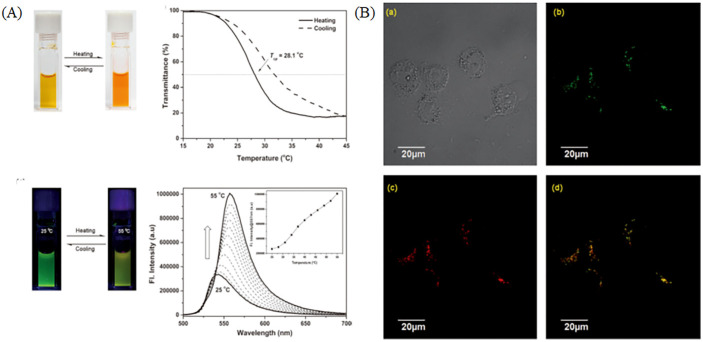
(**A**) Pictures and spectra of BDP-1 in diverse temperature solutions. (**B**) The staining operations and co-localization pictures of HeLa cells stained with BDP-1 and LTR (a commercial lysosomal probe). DIC pictures (**a**) of HeLa cells; Confocal pictures of HeLa cells stained with BDP-1 (**b**) and LTR (**c**); (**d**) Merged pictures of (**b**) and (**c**). (**b**) Confocal pictures of BDP-1 Reproduced from [115] with permission of the Wiley, copyright 2014.

The lysosomal temperature would be changing due to the function of digesting substance. Kuang et al. have developed a lysosomal targeting fluorescent thermometer with BODIPY structure, and it was used to visualize lysosomes [115]. The probe is sensitive to temperature, and it shows color changes and fluorescence changes when the temperature varied (Figure 24A). In the co-staining experiment, the probe overlapped well with LTR (Lyso-Tracker Red) with the overlapping percentage of 88%, indicating the targeting of lysosomes (Figure 24B). Recently, multifunctional probes for the temperature were developed. Wang et al. have exploited a lysosome-targeting nanosensor for visualizing intracellular pH values and temperature, and the nanosensor was used to visualize lysosomes due to the pH (3.0–9.0) and temperature response range (20 to 60 °C) [116]. Organic probes for visualizing temperature were rare, and the multifunctional temperature-sensitive probe would be the tendency in the future.

### 5.3. Organic Probes for Cell Membrane Temperature

The lipid composition of cells (cell membrane etc.) would be adjusted when the ambient temperature changes, so monitoring cell membrane temperature changes is of vital importance to study lipid composition [117,118]. In 1976, VAGELO et al. used a novel fluorimetric probe (β-Parinaric Acid) to measure the effects of temperature changes on membranes [119]. However, they did not use the probe to visualize the temperature changes on cell membrane [119]. To the best of our knowledge, there are no more probes suitable for visualizing changes of cell membrane temperature. The organic probes for visualizing cell membrane temperature changes are quite rare, and the relevant probes are still in exploitation.

### 5.4. Organic Probes for Endoplasmic Reticulum Temperature

**Figure 25:**
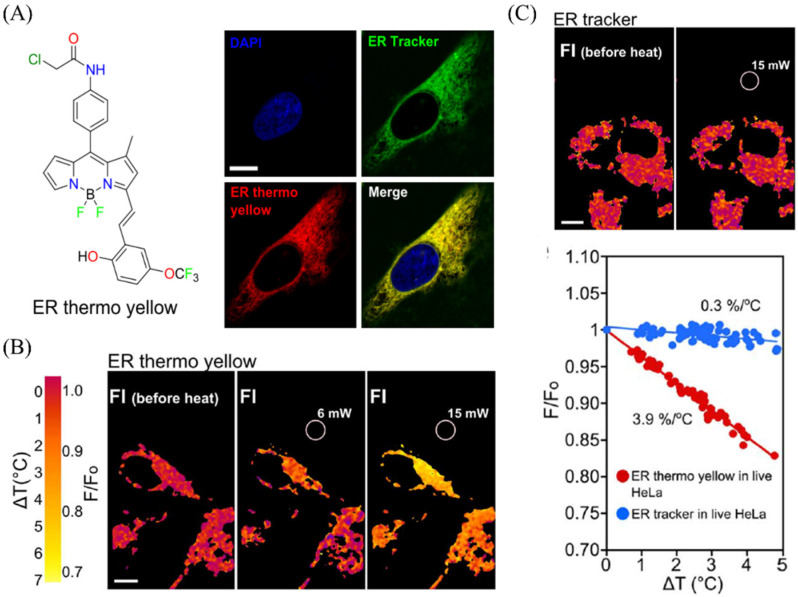
(**A**) the molecular structure of ER probes (ER thermos yellow) for visualizing ER in HeLa cells. (**B**) The temperature pictures of ER thermos under diverse laser powers (6 mW,15 mW). (**C**) The temperature pictures of ER tracker under 15 mW laser powers, and the corresponding data. Reproduced from [120] with permission of the Springer Nature, copyright 2014.

Endoplasmic reticulum (ER) temperature is one of the most important parameters in ER, so visualizing the ER temperature is significant. Chang group have reported an endoplasmic reticulum probe with temperature-sensitive properties for visualizing ER temperature changes in HeLa cells [120]. The ER thermos yellow could target ER (Figure 25A), and it could be used to evaluate the temperature changes in live HeLa cells while the commercial ER probe (ER tracker) did not have the properties (Figure 25B,C). The probes for visualizing the ER temperature are relatively rare, and the research in this area is continuing.

## 6. Conclusions and Outlook

The fluorescence probes for visualizing microenvironment changes in living cells and tissues could provide a useful method for biology and pathology, and it would promote the development of biology. In this review, we summarize recent discoveries in the development of microenvironment-sensitive probes. Some representative examples were displayed and classified to their sensitive microenvironment categories. We highlighted the basic design strategies, fluorescence spectra and the biological applications of these microenvironment-sensitive probes.

Though great progress has been achieved for microenvironment-sensitive probes, there are also some probes need to be exploited in this area. For example, the reversible ratiometric organic probes still need to be exploited, though the “off−on” probes could solve some problems. In contrast to the organic fluorescent probes for monitoring micro-environments in an irreversible “off−on” mode, the reversible ratiometric organic probes are more convenient in imaging applications. Ratiometric organic probes enable real-time and in-situ visualization of the changes of micro-environments in dual colors, and this kind of probe could be applied for background-free bio-imaging and quantitative analysis. Moreover, the errors of instrumental fluctuations and staining operation could be eliminated by the ratiometric mode. So, reversible rationmetric organic probes will become the research focus, and more probes in this category would be exploited for visualizing and monitoring micro-environment in living cells and tissues in the near future. In addition, the single probe for dual-color monitoring two specific mico-environments and the single probe for ratiometric monitoring the same mico-environment in two organelles are still rare. Therefore, these probes would be exploited for the visualization of mico-environment in organelles, and they would prompt the development of biology in the future.

In summary, fluorescence probes for visualizing microenvironments have been a powerful tool for the research of organelles, and more probes would be exploited in the near future. We anticipated this review would promote the research of organelle-related disease and denote to motivate the cancer treatment and biological areas (apoptosis or autophagy).

## Data Availability

Not applicable.
